# Dangerous Physicians

**Published:** 1885-07

**Authors:** 


### Editorial.

---

### Dangerous Physicians.

The Sanitary Engineer calls attention to the possibility of the causation of diseases through a disregard of well-known precautions on the part of physicians to prevent the conveyance of contagions from one family to another; although it is now a well-recognized rule for physicians to avoid labor cases when they have cases of puerperal fevers in their practice, we fear it is true that many physicians "will go directly from a case of scarlet fever to another house containing children, without taking any special precautions."

In this connection, we would call attention to the experiments of Dr. Wassing, as published in the *Archiv. für Hygiene*, and of Prof. Foster in the *Centralblatt für Klinische Medicin*, upon which the *Maryland Medical Journal* makes the following comments:

"These experiments show conclusively that the ordinary methods of cleansing the hands with soap and water are not efficacious in rendering those members aseptic. Solutions of various strengths of carbolic acid, boracic acid, zinc and iron chlorides produce no better effect. The tip of the finger dipped in sterilized culture fluids, after this treatment, invariably caused the development of bacteria in from twenty-four to sixty hours. Immersions in sublimate solutions of 1 to 1000 or 2000, after washing with soap and water, were found, however, to leave the hands '*fairly free*' and safe. These experiments should have the widest possible circulation among the laborers in the medical and surgical field, and the lessons they teach taken very seriously to heart. They show that we are in constant danger of carrying death in our hands instead of healing, and that by the exercise of a very simple precaution we may avoid the danger. The late Professor Meigs, in his controversy with Professor Holmes upon the contagiousness of puerperal fever, said: 'The physician should be a gentleman, and *gentlemen* should have clean hands.' But there is a broad line to be drawn between cleanliness as seen from the 'gentleman's' point of view and that at which the medical man should aim. It is true that most of the varieties of bacteria which we are shown to carry around under our finger nails are, probably, harmless, but the knowledge that we may with equal facility convey those which produce puerperal fever, erysipelas, and other septic conditions, should make us appreciate our responsibilities and keep us keenly alive to the preventive means thrown in our way. If we know anything about germs and the means of destroying them, we know that a solution of corrosive sublimate, 1 to 1000 or 1 to 2000, is a germicide of the most reliable character, and what could be simpler than washing the hands in such a solution after participating in a post-mortem examination or handling anything suspicious? The slowness with which the sublimate dissolves and the consequent delay, may be avoided by using the antiseptic tablets suggested by Dr. Chas. Meigs Wilson in a letter to the Philadelphia *Medical News* of December 20, 1884, and practically prepared by Messrs. John Wyeth & Bro. These tablets contain each 7.3 grs. of sublimate and 7.7 grs. ammonium chloride—the latter being used to increase the solubility of the former. One in a pint of water makes a solution of 1 to 1000, and if water is used it dissolves in a very few moments."

